# International real-world study on osilodrostat efficacy and safety in adrenal Cushing syndrome

**DOI:** 10.1210/clinem/dgag115

**Published:** 2026-03-13

**Authors:** Marta Araujo-Castro, Irina Bancos, Mario Detomas, Martin Reincke, Mahdi Salehi, Haibo Lu, Barbara Altieri, Markus Kroiss, Matthias Oettle, Fernando Guerrero-Pérez, Felicia A Hanzu, Rogelio García-Centeno, Laura Gónzalez-Fernandez, María Pasarón, Paola Gracia Gimeno, Lucía Manzano Valero, Ana Castro Luna, Ana Irigaray Echarri, María Dolores Ollero Garcia-Argullo, Wilfredo Antonio Rivera Martínez

**Affiliations:** Endocrinology and Nutrition Department, Hospital Universitario Ramón y Cajal, Madrid 28034, Spain; Instituto de Investigación Biomédica Ramón y Cajal (IRYCIS), Madrid 28034, Spain; Division of Endocrinology, Diabetes, and Nutrition, Mayo Clinic, Rochester, MN 55905, USA; Department of Internal Medicine I, Division of Endocrinology and Diabetes, University Hospital, University of Würzburg, Würzburg 97080, Germany; Department of Medicine IV, LMU University Hospital, LMU Munich, Munich 81377, Germany; Division of Endocrinology, Diabetes, and Nutrition, Mayo Clinic, Rochester, MN 55905, USA; Luoyang Key Laboratory of Clinical Multiomics and Translational Medicine, Key Laboratory of Hereditary Rare Diseases of Health Commission of Henan Province, Henan Key Laboratory of Rare Diseases, Endocrinology and Metabolism Center, The First Affiliated Hospital, and College of Clinical Medicine of Henan University of Science and Technology, Luoyang 471003, China; Department of Internal Medicine I, Division of Endocrinology and Diabetes, University Hospital, University of Würzburg, Würzburg 97080, Germany; Department of Medicine IV, LMU University Hospital, LMU Munich, Munich 81377, Germany; Department of Medicine IV, LMU University Hospital, LMU Munich, Munich 81377, Germany; Department of Endocrinology and Nutrition, Bellvitge University Hospital-IDIBELL, L’Hospitalet de Llobregat, Barcelona 08907, Spain; Biomedical Research Institute of Bellvitge (IDIBELL), L′Hospitalet de Llobregat, Barcelona 08907, Spain; Endocrinology and Nutrition Department and Institut de Investigacions Biomediques Pi I Sunyer Barcelona, Hospital Clínic of Barcelona, University Barcelona, Barcelona 08036, Spain; Endocrinology and Nutrition Department, Hospital Universitario Gregorio Marañón, Madrid 28007, Spain; Endocrinology and Nutrition Department, Hospital Universitario Gregorio Marañón, Madrid 28007, Spain; Endocrinology and Nutrition Department, Hospital Universitario de Cabueñes, Gijón (Asturias) 33203, Spain; Endocrinology and Nutrition Department, Hospital Universitario Royo Villanova, Zaragoza 50015, Spain; Endocrinology and Nutrition Department, Hospital Universitario Toledo, Toledo 45007, Spain; Endocrinology and Nutrition Department, Hospital Universitario Toledo, Toledo 45007, Spain; Endocrinology and Nutrition Department, Hospital Universitario Navarra, Pamplona 31008, Spain; Endocrinology and Nutrition Department, Hospital Universitario Navarra, Pamplona 31008, Spain; Endocrinology Department, Clínica Imbanaco, Cali 760042, Colombia

**Keywords:** adrenocortical carcinoma, osilodrostat, urinary free cortisol, Cushing syndrome, adrenal adenoma

## Abstract

**Context:**

Most of the patients included in the clinical trials and real-world studies with osilodrostat only include patients with adrenocorticotropic hormone dependent Cushing syndrome (CS), while data on the efficacy and safety of osilodrostat in patients with adrenal CS is scarce.

**Objective:**

To assess the efficacy and safety of osilodrostat in adrenal CS.

**Methods:**

International study of patients with adrenal CS: patients treated with osilodrostat at any time were enrolled in the safety evaluation and those treated for longer than 4 weeks, in the efficacy evaluation. Patients were classified as responders if they experienced a reduction in urinary free cortisol (UFC) >50% (complete responders when UFC levels were below the upper limit of normal [ULN] and partial responders if there was a reduction >50% but not normalization).

**Results:**

Twenty-eight patients with adrenal CS were enrolled: 16 with adrenocortical carcinoma and 12 with benign disease. Osilodrostat was used in monotherapy in 22 patients and in combination with metyrapone in 6 cases. In those patients treated for longer than 4 weeks (n = 21), 66.7% were classified as responders (28.6% with complete response and 38.1% with partial response), and for those treated for longer than 12 weeks, the rate of response increased to 87.5% The use of osilodrostat as a nonfirst-line therapy (odds ratio 15.0, *P* = .010) was a predictor of response. Osilodrostat led to a significant decrease in systolic blood pressure and body weight (*P* < .05). Nine patients developed one or more adverse events and in 56% (n = 5) led to osilodrostat discontinuation.

**Conclusion:**

Osilodrostat controls hypercortisolism in 66.7% of patients with adrenal CS treated for longer than 4 weeks and in 87.5% of cases treated for longer than 12 weeks, with a positive impact on blood pressure and body weight. Patients who received osilodrostat after other previous steroidogenesis inhibitors have a higher probability of response.

Endogenous Cushing syndrome (CS) is a severe endocrine disorder caused by chronic exposure to hypercortisolism and is associated with a wide range of metabolic, cardiovascular, immune, thrombotic, and neuropsychiatric morbidities (1). Based on the underlying mechanism, CS can be broadly classified into 2 main etiological categories: adrenocorticotropic hormone (ACTH)-dependent CS, which results from excessive secretion of ACTH, and ACTH-independent CS, in which cortisol excess arises directly from the adrenal gland (2).

The cornerstone of treatment for adrenal CS is surgical removal of the cortisol-secreting adrenal lesion (3). However, medical therapy also plays an important role in several clinical situations, including patients who are not candidates for surgery, those who decline surgical treatment, cases in which complete tumor resection cannot be achieved because of metastatic disease or residual tumor, and situations in which temporary control of hypercortisolism is required while awaiting surgery (4). Several steroidogenesis inhibitors are currently available for the medical management of CS (5). Nevertheless, some patients do not tolerate these treatments, and others present with severe hypercortisolism that requires rapid and effective reduction of cortisol levels. In such situations, additional therapeutic options are needed to achieve adequate biochemical control of hypercortisolism (6). Osilodrostat is an oral imidazole derivative that inhibits 11β-hydroxylase, the enzyme responsible for the final step of cortisol synthesis in the adrenal cortex. By inhibiting this enzyme, osilodrostat effectively suppresses cortisol production (7). Most clinical studies evaluating osilodrostat have focused on patients with ACTH-dependent CS, particularly those with Cushing disease (CD) (7-13), while data on the efficacy and safety of osilodrostat in patients with adrenal CS are scarce (12, 14-16). Only a small number of reports have evaluated the use of osilodrostat in adrenal CS, and the response rates described in these studies appear to be lower than those reported in patients with ACTH-dependent disease (12, 14-16). More recently, the LINC 7 study analyzed the efficacy and safety of osilodrostat in patients with adrenocortical carcinoma (ACC, n = 19), adrenal adenomas (n = 17), and bilateral adrenal nodular disease (n = 14), being the largest series reported until the current date, with a total of 50 cases included (17). However, in the intention to treat evaluation (efficacy of the treatment after 12 weeks of osilodrostat therapy), only 19 patients with adrenal CS were analyzed, and the reported efficacy of osilodrostat was quite lower than the described in previous studies (67% of the patients with ACC, 33% with adrenal adenomas, and 86% with bilateral adrenal disease had a complete biochemical response). These observations raise the question of whether the efficacy and safety profile of osilodrostat differs between ACTH-dependent and ACTH-independent forms of CS.

Therefore, the aim of the present study was to evaluate the efficacy and safety of osilodrostat in patients with adrenal CS, including both benign and malignant etiologies, and to explore potential predictors of treatment response in this population.

## Materials and methods

### Study design and definitions

This was a multicenter, retrospective cohort study (international adrenal CS database) that included a total of 433 patients with adrenal CS (251 with unilateral adrenal disease, 87 with bilateral disease, and 95 with ACC) from multiple centers (23 centers in Spain, 1 in Croatia, 2 in Germany, 10 in Colombia, 1 in the United States, and 1 in Italy). Patients treated with osilodrostat were managed in 10 centers: 7 in Spain, 2 in Germany, and 1 in the United States.

Patients were followed by endocrinologists working in specialized endocrinology departments at the participating hospitals. The protocol for the use of osilodrostat (including the initial dose, dose titration, and the use of block-and-replace therapy [B&R], among other strategies) was determined according to the clinical experience of the treating physicians.

The diagnosis of CS was based on current clinical practice guidelines (18). The inclusion criteria to enter in the study were as follows: (1) a biochemically confirmed diagnosis of CS of adrenal origin, (2) treatment with osilodrostat at any time (at least one dose), and (3) availability of data on efficacy or safety. We have also included those patients who developed adverse events (AEs) after osilodrostat initiation with no available UFC after treatment, with a goal to avoid biases on the analysis of safety. For the efficacy assessment, we only included patients treated with osilodrostat for longer than 4 weeks.

Severe CS was defined by urinary free cortisol (UFC) levels above 5 times the upper limit of normal (ULN) or associated with severe and life-threatening complications of hypercortisolism (6). The clinical classification of ACC was based on the TNM-ENSAT staging system (I-IV) (19). For benign and malignant adrenal tumors, we also reported previous treatments used for tumor and hypercortisolism control (Table S1) (20).

The diagnosis of hypertension was based on a previous history of hypertension or treatment with antihypertensive medication, and type 2 diabetes mellitus diagnosis was established for those with previous history of diabetes or who were treated with antihyperglycemic medication.

The study was approved by the Ethics Committee of the Hospital Universitario Ramón y Cajal, Madrid, Spain (approval date: November 26, 2024, code: ACTA 472) and in each collaborating center. The waiver of informed consent was approved by the Ethics Committee due to the retrospective nature of the study. Informed consent was requested only for patients who continued follow-up.

### Outcomes: efficacy and safety

Urinary free cortisol was measured by chemiluminescent immunoassay or mass spectrometry and radioimmunoassay. As the normal range of UFC differed across centers, we calculated the deviations above the ULN for each UFC value (UFC × ULN).

The main efficacy endpoint was the proportion of responders after treatment with osilodrostat for longer than 4 weeks: complete or partial response. Complete response was defined by UFC <ULN or development of adrenal insufficiency during osilodrostat therapy. Partial response was considered as a reduction of UFC >50% but with no UFC normalization. We defined no response as a mean UFC >ULN and with <50% reduction from baseline.

UFC values were recorded after 2, 4, and 12 weeks of treatment and at the last available follow-up visit. However, due to the retrospective nature of the information, UFC was not available for all patients at all these different time points. In addition, we have evaluated the time necessary to achieve hypercortisolism control. Data on morning serum cortisol, late-night salivary cortisol, and ACTH at different time points were available in few cases. Therefore, the analysis of these data was not included in the current study (>60% missing data).

Regarding clinical and biochemical variables, we collected information on systolic and diastolic blood pressure (BP), number of antihypertensive medications, serum potassium levels, body weight (kg), glucose levels (mg/dL), HbA1c (%), total cholesterol, low-density lipoprotein, and high-density lipoprotein at diagnosis of adrenal CS, before osilodrostat initiation and after treatment (after 4 and 12 weeks and at the last available follow-up visit).

In relation to osilodrostat treatment, we collected information on starting, maximum, and maintenance doses (mg/day). Information about the treatment strategy used was recorded: titration (when osilodrostat was used without glucocorticoid replacement therapy), B&R regimen upfront, or initial titration followed by B&R.

Safety evaluations included data on the development of AEs: hyperandrogenism, glucocorticoid withdrawal syndrome (GWS), adrenal insufficiency, hypertension, hypokalemia, QT interval prolongation, edema, or other AEs. We also described the proportion of patients who discontinued the drug due to the development of AEs.

### Statistical analysis

The statistical analysis was performed with STATA 15. Categorical variables were expressed as percentages and absolute numbers and quantitative variables as mean (±standard deviation) or median (range), depending on whether the assumption of normality was met. The Wilcoxon signed-rank test was used for comparison of variables of UFC, clinical score, systolic BP, diastolic BP, body weight, and the biological parameters, such as potassium and glucose, glycemia, and HbA1c before and during osilodrostat therapy. In all cases, a 2-tailed *P*-value <.05 was considered statistically significant. Univariate and multivariate logistic regression analysis was used to identify predictors of response to osilodrostat therapy. Correlation between continuous variables was tested by the estimation of the Pearson correlation coefficient (*r*). The Jonckheere–Terpstra trend test was used to analyze whether there were ordered trends in the UFC groups and nonresponse to osilodrostat.

## Results

### Baseline characteristics: at the time of the diagnosis of adrenal CS

Twenty-eight patients with adrenal CS were included in the study: 16 with ACC (13 stage IV and 3 nonmetastatic ACC) and 12 with benign adrenal disease (5 with unilateral adrenal adenoma and 7 with bilateral adrenal disease). The median age of the cohort at the time of the CS diagnosis was 59.5 years (range 27-81), and 68% (n = 19) were women. Regarding ethnicity, 16 were Caucasian, 11 Native American, and 1 Asian. The median UFC at the time of CS diagnosis was 6.3 times above the ULN (range 1.3-53.3), and median ACTH levels were 5 pg/mL (range 1-15). There were 15 patients (53.6%) with severe CS. Patients with ACC were younger, had higher systolic and diastolic BP, had a higher prevalence of hypokalemia and unilateral adrenal tumors, and had more severe hypercortisolism than patients with benign adrenal CS (Table 1).

**Table 1:** Baseline characteristics at diagnosis of Cushing’s syndrome

Variable	ACC (n = 16)	Benign adrenal disease (n = 12)	*P*-value
Clinical and biochemical data			
Age (years)	50.7 ± 13.83	65.7 ± 13.12	.008
Female sex	75% (n = 12)	58.3% (n = 7)	.350
Type 2 diabetes	37.5% (n = 6)	58.3% (n = 7)	.274
Hypertension	87.5% (n = 14)	91.7% (n = 11)	.724
Dyslipidemia	50% (n = 8)	91.7% (n = 11)	.019
Hypokalemia	81.3% (n = 13)	8.3% (n = 1)	<.001
BMI (kg/m^2^)	30.5 ± 5.62	29.6 ± 6.04	.693
Systolic BP (mmHg)	157.5 ± 30.20	132.8 ± 20.12	.025
Diastolic BP (mmHg)	93.6 ± 18.95	80.7 ± 11.68	.054
Fasting plasma glucose levels (mg/dL)	124.3 ± 45.19	127.8 ± 28.02	.831
HbA1c (%)	6.5 ± 1.37	6.5 ± 1.39	.942
Serum potassium levels (mEq/mL)	3.4 ± 0.54	4.1 ± 0.36	.002
Cortisol after dexamethasone suppression test (µg/dL)	26.6 ± 11.54	8.1 ± 7.20	.001
UFC (values above the ULN)	21.1 ± 19.59	3.7 ± 2.72	.016
ACTH (pg/mL)	8.1 ± 15.58	6.0 ± 3.83	.666
DHEAS (µg/dL)	746.6 ± 1022.28	44.7 ± 47.78	.055
Radiological data			
Unilateral disease	93.8% (n = 15)	41.7% (n = 5)	.003
Tumor size of the largest nodule (mm)	152 ± 187.87	28.6 ± 19.21	.082

Abbreviations: ACC, adrenocortical carcinoma; BMI, body mass index; BP, blood pressure; DHEAS, dehydroepiandrosterone sulfate; ULN, upper limit of normal; UFC, urinary free cortisol.

### Osilodrostat: previous treatments

Previous and/or simultaneous treatments before or with osilodrostat are described in Table S1 (20). In summary, in the group of patients with ACC, 9 patients underwent adrenalectomy, 13 received mitotane, 13 chemotherapy, 10 immunotherapy, and 4 radiotherapy. In the group of bilateral benign adrenal disease (n = 7), 2 underwent unilateral adrenalectomy (1 before starting osilodrostat and 1 after starting osilodrostat), and of the 6 patients with unilateral disease, 4 patients underwent unilateral adrenalectomy after osilodrostat treatment. Of the 13 patients with ACC treated with mitotane, mitotane was used in combination with osilodrostat in 4 patients.

In relation to the previous treatments used for hypercortisolism control before osilodrostat, 9 were treated with metyrapone, 4 with ketoconazole, and 4 with ketoconazole in combination with metyrapone. In summary, osilodrostat was used as the first-line therapy in 15 patients, as a second-line therapy in 9 (in 2 of them in combination with metyrapone), as a third-line therapy in 3, and as a fourth-line therapy in 1 case (Table S2) (20).

### Efficacy of osilodrostat

Overall, the median duration of the therapy with osilodrostat was 7 months (range 1-64). Osilodrostat was used in monotherapy in 22 patients and in combination with metyrapone in 6 cases. The median starting dose was 4 mg/day (range 1-10), the maintenance dose was 9 mg/day (range 1-50), and the maximum dose was 10 mg/day (range 1-50). No significant differences were detected in the initial (*P* = .910) or maximum doses (*P* = .412) when osilodrostat was used in monotherapy or in combination. The initial, maintenance and maximum doses employed in each patient according to the UFC level at diagnosis and before starting osilodrostat are described in Table S2 (20). In the group of combination therapy, the median dose of metyrapone was 4 g/day (range 2-4.5). The median UFC (×ULN) before starting osilodrostat was 4.2 (0.6-53.3). In this regard, 1 patient had normal UFC (0.6× ULN) at the time of osilodrostat initiation. The diagnosis of hypercortisolism in this case was based on clinical data (CS phenotype and hypokalemia) and high levels of baseline and night serum cortisol (23.4 and 24.6 μg/dL, respectively). This patient was not included in the efficacy evaluation as UFC was normal at the time of osilodrostat initiation.

A positive correlation was observed between UFC before osilodrostat initiation and the initial (*r* = 0.41, *P* = .032) and maximum doses (*r* = 0.53, *P* = .005) of osilodrostat. The maintenance doses (at last visit) were significantly higher than the starting doses (12 ± 11.87 vs 4.3 ± 2.84 mg/day, *P* = .002), but no significant differences were detected between the maintenance and maximum doses (*P* = .622). No correlation was detected between the initial (*r* = −0.18, *P* = .411) or maximum doses (*r* = −0.04, *P* = .864) and the percentage decrease in UFC with osilodrostat.

There were 10 patients treated with osilodrostat using the titration approach followed by B&R, and 1 patient in the combination therapy who started B&R from the beginning. Overall, B&R was used in 4 of the 6 patients treated in combination with metyrapone and in 6 of the 22 patients treated with monotherapy. Hydrocortisone was the glucocorticoid of choice in 9 patients (median dose 20 mg/day), while 1 patient was treated with dexamethasone (2 mg/day). The maximum doses of osilodrostat were higher in the B&R group than in the titration group (19.2 ± 13.24 vs 10.3 ± 9.25 mg/day, *P* = .050).

**Figure 1:**
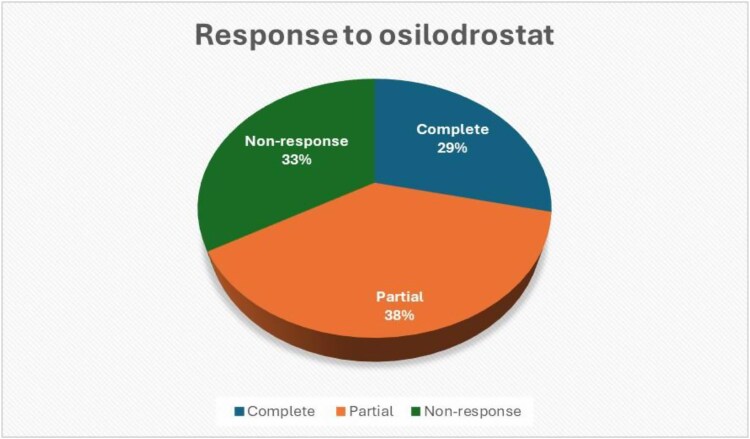
Type of response to osilodrostat therapy in 21 patients with adrenal Cushing syndrome.

Of the 28 patients treated with osilodrostat, 2 patients withdrew treatment before hormonal assessment due to poor tolerability, 1 died after only 1 month of therapy precluding UFC assessment, 3 patients were treated for less than 4 weeks, and 1 patient had normal UFC levels at the time of osilodrostat initiation (UFC 0.6× ULN). Thus, only 21 patients were included in the efficacy assessment (treatment with osilodrostat for longer than 4 weeks). The response to osilodrostat was 66.7% (n = 14): 6 with complete response and 8 with partial response. The rate of response was 80% in the combination therapy group and 62.5% in the monotherapy group (Fig. 1). In the group of complete responders, the median time to normalize UFC was 10 weeks (range 5-17). Response tended to be higher in patients with benign disease than in those with ACC (87.5% vs 53.9%, *P* = .112).

The rate of response increased to 87.5% (62.5% with complete response and 25% with partial response) when we assessed those cases treated with osilodrostat for longer than 12 weeks (n = 7/8).

**Figure 2:**
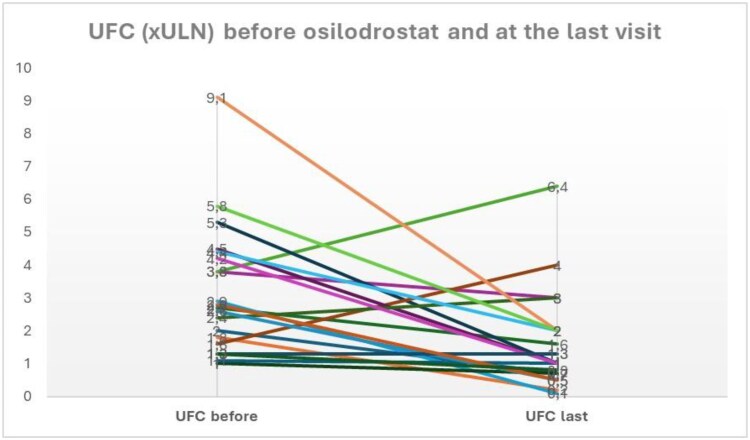
Urinary free cortisol response to osilodrostat in patients with adrenal Cushing’s syndrome. ULN, upper limit of normal.

The evolution of UFC in the different times for those patients treated for longer than 1 month (before, after 2 weeks, 1 month, 3 months, and at the last visit) is described in Fig. 2. Three patients had a small increase in UFC from baseline to >ULN at last visit, and all of these cases had tumoral progression associated with the UFC increase.

### Predictors of osilodrostat response

When we compared the group of responders (n = 14) and nonresponders (n = 7), we found that the probability of response was higher when osilodrostat was used as a second-/third- or fourth-line therapy (odds ratio [OR] 15, 95% confidence interval [CI] 1.34-167.64, *P* = .010) (Table 2). The use of osilodrostat as a first-line therapy tended to be more common in patients with ACC than benign adrenal CS (68.8% vs 33.3%, *P* = .063). In this regard, the association between response to osilodrostat and the nonfirst-line therapy use disappeared after adjusting by the variable ACC (adjusted OR 11.53, 95% CI 0.97-136.75, *P* = .053).

**Table 2:** Predictors of response to osilodrostat in patients with adrenal Cushing’s syndrome

Variable	Responders (n = 14)	No responders (n = 7)	OR (95% CI), *P*-value
Age (years)	61 ± 13.92	52.9 ± 15.51	1.04 (0.97-1.12), .214
Female sex	71.4% (n = 10)	57.1% (n = 4)	0.53 (0.08-3.54), .516
Type 2 diabetes	64.3% (n = 9)	28.6% (n = 2)	4.50 (0.63-32.29), .118
Hypertension	92.9% (n = 13)	85.7% (n = 6)	2.17 (0.12-40.81), .609
Dyslipidemia	64.3% (n = 9)	71.4% (n = 5)	0.72 (0.10-5.17), .742
Hypokalemia	57.1% (n = 8)	57.1% (n = 4)	1.00 (0.16-6.25), 1.000
Obesity	57.1% (n = 8)	28.6% (n = 2)	3.33 (0.47-23.47), .210
Ethnicity (Caucasian vs others)	57.1% (n = 8)	71.4% (n = 5)	0.53 (0.08-3.76), .521
ACC diagnosis	50% (n = 7)	85.7% (n = 6)	0.16 (0.02-1.77), .096
UFC (×ULN) at diagnosis	11.3 ± 15.90	23.8 ± 18.67	0.96 (0.90-1.02), .164
UFC (×ULN) before osilodrostat	9.3 ± 15.93	21.1 ± 17.23	0.96 (0.91-1.01), .132
UFC <2× ULN before osilodrostat	42.9% (n = 6)	14.3% (n = 1)	4.50 (0.42-47.99), .171
UFC <5× ULN before osilodrostat	71.4% (n = 10)	28.6% (n = 2)	6.25 (0.84-45.57), .059
ACTH (pg/mL)	9.0 ± 16.33	5.0 ± 3.26	1.04 (0.90-1.20), .484
Duration of treatment (months)	15.9 ± 19.95	7.3 ± 8.26	1.06 (0.93-1.20), .230
Initial doses (mg/day)	4.1 ± 2.37	5.4 ± 3.55	0.84 (0.60-1.17), .289
Maximum doses (mg/day)	14.9 ± 13.12	15.5 ± 10.19	1.00 (0.92-1.08), .921
Combination therapy (different than mitotane)	28.6% (n = 4)	14.3% (n = 1)	2.40 (0.21-24.82), .454
Concomitant therapy with mitotane	50% (N = 3)	0%	OR not calculable, .064
Use as nonfirst-line	71.4% (n = 10)	14.3% (n = 1)	15.0 (1.34-167.64), .010

Abbreviations: ACC, adrenocortical carcinoma; UFC, urinary free cortisol; ULN, upper limit of normal.

Those cases with UFC below 5× ULN before osilodrostat initiation tended to have a higher rate of response (OR 6.25, 95% CI 0.84-46.57, *P* = .059). All patients with ACC treated with osilodrostat in combination with mitotane had response, while only 37.5% of the cases treated with osilodrostat without concomitant mitotane (*P* = .064). In addition, it was found that patients with type 2 diabetes tended to have a higher response to osilodrostat than those with no diabetes (81.8% vs 50%, *P* = .122).

**Figure 3:**
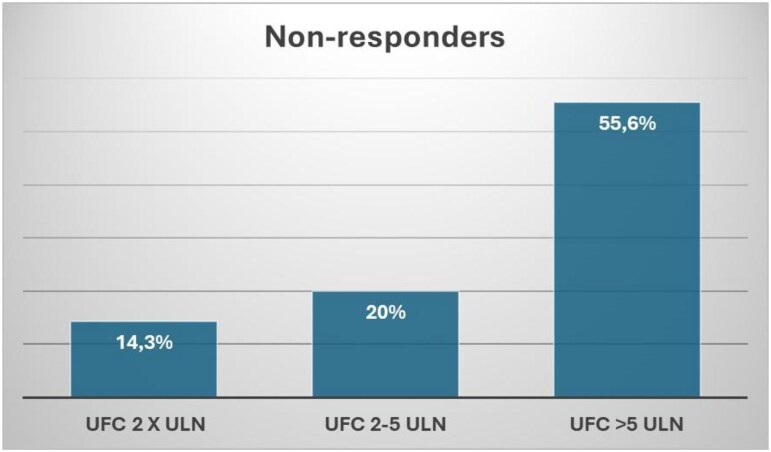
Proportion of nonresponders to osilodrostat according to UFC levels before the initiation of the therapy. A positive tendency to increase the proportion of nonresponders was observed as higher the category was (UFC <2× ULN, UFC 2-5× ULN and UFC >5× ULN) (12.5%, 33.3%, and 55.6%, respectively, MH test for linear trend: χ^2^(1) = 3.32 [*P* = .067]).

In the tendency analysis, patients were stratified into 3 groups based on UFC levels prior to the initiation of osilodrostat (UFC <2× ULN, UFC 2-5× ULN, and UFC >5× ULN). The proportion of nonresponders tended to increase as the category increased (UFC <2× ULN, UFC 2-5× ULN, and UFC >5× ULN) (14.3%, 20%, and 55.6%, respectively, MH test for linear Trend: χ^2^(1) = 3.01 [*P* = .083]) (Fig. 3).

### Impact of osilodrostat on comorbidities

At the time of diagnosis of CS, hypertension was present in 89.3% (n = 25) of patients, dyslipidemia in 67.9% (n = 19), obesity in 50% (n = 14), hypokalemia in 50% (n = 14), and diabetes mellitus in 46.4% (n = 13) (Table 1). The mean levels before the initiation of osilodrostat and at the last available visit are described in Table 3. Overall, most cardiovascular and metabolic-related parameters remained stable during treatment, while a significant decrease of systolic BP and body weight was observed after osilodrostat initiation. In patients with systolic BP >140 mmHg at baseline (39.1%, n = 9/23), 62.5% had systolic BP ≤130 mmHg at the last visit. Among those with diastolic BP >90 mmHg at baseline (34.8%, n = 8/23), 57.1% had diastolic BP ≤90 mmHg at the last visit.

**Table 3:** Impact of osilodrostat treatment on cardiometabolic profile

Variable	Before osilodrostat	After osilodrostat (last available)	*P*-value
FPG (mg/dL)	128.3 ± 46.27 (n = 25)	114.6 ± 45.68 (n = 20)	.286
HbA1c (%)	6.4 ± 1.36 (n = 25)	6.4 ± 1.61 (n = 12)	.844
Total cholesterol (mg/dL)	176.3 ± 42.04 (n = 26)	179.6 ± 56.26 (n = 14)	.346
LDL (mg/dL)	99.6 ± 38.78 (n = 25)	95.6 ± 41.27 (n = 14)	.346
HDL (mg/dL)	53.4 ± 14.50 (n = 22)	48.2 ± 16.62 (n = 11)	.721
Weight (kg)	81.2 ± 18.57 (n = 27)	77.8 ± 20.77 (n = 22)	.009
SBP (mmHg)	137.5 ± 17.55 (n = 28)	123.7 ± 22.33 (n = 32)	.047
DBP (mmHg)	83.2 ± 13.71 (n = 23)	78.9 ± 23.50 (n = 22)	.821
Antihypertensive drugs (n)	2 (0-4) (n = 34)	2 (0-4) (n = 33)	.622
Potassium (mmol/L)	4.1 ± 0.65 (n = 27)	3.9 ± 0.56 (n = 25)	.100

Comparison of cardiometabolic parameters before and after the initiation of osilodrostat, considering the minimum values achieved during the follow-up period. The Wilcoxon signed-rank test was used for comparison of variables.

Abbreviations: DBP, diastolic blood pressure; HDL, high-density lipoprotein; FPG, fasting plasma glucose; HbA1c, glycated hemoglobin A1c; LDL, low-density lipoprotein; SBP, systolic blood pressure.

### Safety of osilodrostat and follow-up

There were 9 patients (32.1%) who developed one or more AEs potentially related to osilodrostat therapy and in 56% (n = 5) of them osilodrostat was discontinued for this reason (1 with adrenal insufficiency, 3 with gastrointestinal discomfort, and 1 gastrointestinal symptoms, brain fog, memory issues, slurred speech, blurry vision, and weakness) (Table 4). None of the AEs were classified as severe. No cases of hyperandrogenism worsening or QT interval prolongation were reported. No significant differences in the initial or maximum doses were observed between patients who developed AEs and those who did not (*P* > .05). The rate of AEs tended to be higher in patients treated for a period shorter than 4 weeks (50%) vs those treated for a longer period (27.3%, *P* = .291) since some of the patients of the first group discontinued the drug due to the development of AEs.

**Table 4:** Adverse events reported with osilodrostat therapy

Adverse event	Proportion (n)
Gastrointestinal intolerance	14.3% (4)
GWS	7.1% (2)
Edema	7.1% (2)
Hypertension	7.1% (2)
Hypokalemia	3.6% (1)
Liver function alteration	3.6% (1)
Hypocortisolism	7.1% (2)
Others	10.7% (3)^a^

Abbreviation: GWS, glucocorticoid withdrawal syndrome.

^*a*^One patient reported brain fog, memory issues, slurred speech, blurry vision, and weakness, another tachycardia, and another hyporexia.

The median follow-up time (from diagnosis of CS to last visit) was 1.5 years (0.2-10.9), being significantly higher in patients with benign disease than in those with ACC (4.4 ± 3.5 vs 1.4 ± 1.32 years, *P* = .004). At the last follow-up, 16 patients had died (9 due to tumor progression, 3 due to hypercortisolism complications, and 4 due to other medical or unknown causes). All the patients who died had ACC except 1 patient with benign adrenal disease who died due to progression of other nonadrenal tumor.

Overall, at the last visit, only 2 of the 6 patients in combination with metyrapone remained on osilodrostat, while 3 died due to tumor progression and 1 developed adrenal insufficiency. In the group of monotherapy, only 6 continued on osilodrostat, while 72.7% (n = 16) discontinued. The reasons for discontinuation were cure after surgery (n = 3), death (n = 8), and development of AEs (n = 5).

## Discussion

Our study is the first international study specifically focused on evaluating the efficacy and safety of osilodrostat in patients with adrenal CS, including benign and malignant tumors. The main findings of our study were that osilodrostat led to a normalization of UFC or a decrease >50% in 67% of these patients and had a positive impact on BP control and body weight. In addition, the incidence of AEs was relatively low, with adrenal insufficiency occurring only in 7% of the patients, which is much lower than the reported incidence when osilodrostat is used for the treatment of CS of other etiologies (9, 21). As a novelty of our study, we found that the use of osilodrostat as a nonfirst-line medical option for hypercortisolism control might be a potential predictor of response to osilodrostat. However, this association seemed to be influenced by the more frequent use of osilodrostat as a nonfirst-line therapy in patients with benign adrenal disease than in cases with ACC, as the association disappeared after adjusting for the presence of ACC.

In relation to the efficacy of osilodrostat, we observed some differences when we compared our results with those described in patients with ACTH-dependent CS. In our study, 67% of the cases were classified as responders and only 28.6% as complete responders. Nevertheless, the rate of control increased to 87.5% when we analyzed those patients treated with osilodrostat for longer than 12 weeks. In this regard, it should be highlighted that the duration of the treatment is an important factor to consider as for cases with moderate-severe CS, several titrations of the doses are frequently needed to get the correct dose that controls hypercortisolism. Although the rate of control is similar to that described with other steroidogenesis inhibitors such as ketoconazole (22) and metyrapone (23), the response to osilodrostat in adrenal CS in our series was lower than that previously reported in patients with CD (13, 16, 24) and ectopic CS (ECS) (7, 25) in a similar scenario of real-world practice. For example, in our recent Spanish studies, we reported UFC normalization in 88% (n = 14/16) of ECS patients (25) and in 89.2% of patients with CD (13), while no cases of nonresponse were identified. However, in the recent LINC 7 study evaluating the effectiveness of osilodrostat in nonpituitary CS in real-world practice, the results were in accordance with ours (17). The rate of complete response in the LINC7 was 44.2% (95% CI 30.5-58.7), with 41% in ECS (n = 12/29), 66.6% in ACC (n = 4/6), 33.3% in adrenal adenomas (n = 1/3), and 85.7% in bilateral adrenal disease (n = 6/7). It should be noted that in the LINC7 study, only 6 patients with ACC and 10 patients with benign disease were included in the effectiveness evaluation, so the results should be interpreted with caution. On the other hand, in the Tabarin et al’s series with 7 patients with ACC included, osilodrostat normalized UFC in all patients (12). The explanation for the differences across studies is not fully understood, as neither the severity of CS nor other factors that differ across series have been described as predictors of response to osilodrostat in previous studies. Nonetheless, considering all the data together, it seems that osilodrostat is a less effective treatment for patients with adrenal CS than for ACTH-dependent CS, and the real explanation for this finding is unknown.

One of the most important findings of our study is that the use of osilodrostat after the use of other previous adrenal steroidogenesis inhibitors (ie, as a second-/third- or fourth-line therapy vs first line) might be a potential predictor of response to osilodrostat. The higher efficacy of osilodrostat when it was used in a nonfirst-line therapy may be related to the more frequent use of osilodrostat as first-line therapy in patients with ACC than with benign adrenal CS and a tendency to a higher proportion of patients treated with concomitant mitotane in the group of nonfirst-line therapy in comparison with the first-line therapy group. In this regard, although the differences were not statistically significant, our results suggest that ACC patients showed a worse control of hypercortisolism than benign adrenal CS, and patients with ACC treated with concomitant mitotane had a higher rate of response than those treated without mitotane. The lack of statistically significant differences may be due to a type II error or a small sample size among the comparators, especially considering the relationship that we found between ACC and severe hypercortisolism, a predictor of lower response found in the statistical analysis. In addition, we identified 3 patients with ACC who experienced UFC increase during osilodrostat therapy in the context of tumoral progression. It is known that cortisol hypersecretion is an independent poor prognostic parameter of ACC, and the normalization of hormonal excess represents a priority in the management of patients with hormone-secreting ACC (26). Regarding the severity of hypercortisolism as a predictor of response, the rate of hypercortisolism control tended to be higher in patients with UFC before starting osilodrostat <5× ULN than in those with higher levels. In line with these results, the pooled analysis of LINC 2, 3, and 4 showed that patients with baseline UFC <2× ULN generally achieved UFC control faster than those with UFC 2-5 or >5× ULN, requiring a lower median osilodrostat dose for hypercortisolism control (27).

Hypertension and obesity are common comorbidities in patients with CS and are present in 70-90% of patients at diagnosis, which contributes to the increased morbidity and mortality risk. One of the main outcomes of medical therapy (together with UFC normalization/reduction) is the improvement of comorbidities and quality of life. The most common evolution of the control of the comorbidities in patients with ACC and in CS in general is to worsen over time, especially if hypercortisolism remains uncontrolled. In our cohort, osilodrostat avoided this deterioration and even led to an improvement in the control of systolic BP and body weight. These results are in line with those described in other real-world studies and clinical trials (13, 16, 25, 28). For example, in the pooled analysis of LINC 2, 3, and 4, antihypertensive medication dose was reduced or stopped in 26.8% of patients, and in patients with diabetes, mean fasting plasma glucose levels and HbA1c decreased after osilodrostat therapy initiation (28). We have to highlight that given the life-threatening clinical impact of comorbidities associated with severe hypercortisolism in these patients, the rapid systemic evaluation and multidisciplinary treatment in association with targeted cortisol-lowering therapies are critical for optimizing clinical outcomes (6).

AEs were reported in 32% of the patients in our series, leading to osilodrostat discontinuation in 5 of them, but none of these AEs were classified as severe. In accordance with previous studies, gastrointestinal AEs, including nausea and vomiting, were the most commonly noted (29). Adrenal insufficiency merits a special mention. We observed a lower prevalence of hypocortisolism (7%) than that reported in patients with ACTH-dependent CS (7, 9, 12). For example, in the LINC 3 (patients with CD), adrenal insufficiency occurred in 28% of the patients (9); in the Dormoy et al’s study with 33 patients with ECS included (7), hypocortisolism occurred in 8 (24%) patients; and in the Tabarin et al’s series (12) with 7 patients with ACC included, mild and transient adrenal insufficiency was observed in 43% of the cases (n = 3/7). These differences may be related to the differences in the doses used across studies and/or the baseline characteristics of the cases included. For example, the maximum doses in our study were 10 mg/day (range 1-50), while it was 20 mg/day (range 4-40) in the Tabarin et al’s series (12) and 20 mg/day (10-100/mg/d) in the Dormoy et al’s study (7). However, it was lower in the LINC3 with patients with CD (9), but the population study was very different, as the proportion of cases with severe CS was 71% in Tabarin et al’s study (12) and 91% in the Dormoy et al’s study (7). In this regard, it is important to emphasize that the initial doses of osilodrostat should be tailored based on the severity of hypercortisolism. As we recently proposed in our Spanish Consensus for the management of severe CS, in general for severe hypercortisolism (UFC >5 but <10× ULN), osilodrostat should be employed using higher starting doses (10-30 mg/day), and in patients with life-threatening hypercortisolism (UFC >10× ULN), very high initial doses (30-60 mg/day) are necessary for rapid cortisol control (6). However, in the recent pooled analysis of the LINC 2, 3, and 4, the doses of osilodrostat did not predict the development of adrenal insufficiency, while patients with less severe hypercortisolism at baseline (UFC <2 or 2-5× ULN) and no prior medical therapy had a significantly lower risk of experiencing hypocortisolism-related AE than those with baseline UFC >5× ULN and prior medical therapy (27).

Our study is the first and largest study focused on evaluating the efficacy and safety of osilodrostat and the first study that identified potential predictors of response to osilodrostat in patients with adrenal CS. However, we are aware of some of the limitations of the study. One of the biggest limitations is the retrospective nature of the study with its intrinsic limitations and the variability of the protocols used across participating centers, including different starting doses and the fact that information on UFC and cardiometabolic parameters was not available in all the patients at the different evaluation times. Furthermore, although it is the reported largest study, the sample size is relatively small, so differences between groups may be difficult to detect due to the low power in the statistical analysis. In addition, we also know that chronotherapy is another important aspect that should be considered in the treatment of CS as the administration in the afternoon–evening may be useful to restore the circadian rhythm and improve circadian cortisol profiles, quality of life, and sleep (30). However, in our study, patients were treated according to traditional recommendations for osilodrostat use, without considering higher doses in the evening.

## Conclusions

Osilodrostat effectively controls hypercortisolism in up to 67% of the patients with adrenal CS treated for longer than 4 weeks and in 87.5% of cases treated for longer than 12 weeks, with a positive impact on BP and body weight. Patients who received osilodrostat after previous steroidogenesis inhibitors had a higher probability of response to osilodrostat.

## Data Availability

The data are not publicly available but is available from the authors upon reasonable request.

## References

[1] Pivonello R, Isidori AM, De Martino MC, Newell-Price J, Biller BMK, Colao A. Complications of Cushing's syndrome: state of the art. Lancet Diabetes Endocrinol. 2016;4(7):611‐629. 27177728 10.1016/S2213-8587(16)00086-3

[2] Nieman LK, Castinetti F, Newell-Price J, et al Cushing syndrome. Nat Rev Dis Primers. 2025;11(1):4. 39848955 10.1038/s41572-024-00588-w

[3] Fassnacht M, Tsagarakis S, Terzolo M, et al European Society of Endocrinology clinical practice guidelines on the management of adrenal incidentalomas, in collaboration with the European Network for the Study of Adrenal Tumors. Eur J Endocrinol. 2023;189(1):G1‐G42. 37318239 10.1093/ejendo/lvad066

[4] Puglisi S, Perotti P, Pia A, Reimondo G, Terzolo M. Adrenocortical carcinoma with hypercortisolism. Endocrinol Metab Clin North Am. 2018;47(2):395‐407. 29754640 10.1016/j.ecl.2018.02.003

[5] Guarnotta V, Stigliano A, Terzolo M, Arnaldi G. Management of Cushing's syndrome in patients with adrenocortical cancer: state of the art and future perspectives. Rev Endocr Metab Disord. 2025;26(6):1023‐1035. 40736645 10.1007/s11154-025-09989-y PMC12602599

[6] Araujo-Castro M, García-Centeno R, Aller J, et al Executive summary of the consensus document for the management of severe Cushing's syndrome: consensus document of the Neuroendocrinology Focus Group of the Spanish Society of Endocrinology and Nutrition (SEEN). Endocrinol Diabetes y Nutr. 2025;72(10):501654. 10.1016/j.endien.2025.501654 41419261

[7] Dormoy A, Haissaguerre M, Vitellius G, et al Efficacy and safety of osilodrostat in paraneoplastic Cushing syndrome: a real-world multicenter study in France. J Clin Endocrinol Metab. 2023;108(6):1475‐1487. 36470583 10.1210/clinem/dgac691 PMC10188310

[8] Fleseriu M, Pivonello R, Young J, et al Osilodrostat, a potent oral 11β-hydroxylase inhibitor: 22-week, prospective, phase II study in Cushing's disease. Pituitary. 2016;19(2):138‐148. 26542280 10.1007/s11102-015-0692-z PMC4799251

[9] Pivonello R, Fleseriu M, Newell-Price J, et al Efficacy and safety of osilodrostat in patients with Cushing's disease (LINC 3): a multicentre phase III study with a double-blind, randomised withdrawal phase. Lancet Diabetes Endocrinol. 2020;8(9):748‐761. 32730798 10.1016/S2213-8587(20)30240-0

[10] Gadelha M, Bex M, Feelders RA, et al Randomized trial of osilodrostat for the treatment of Cushing disease. J Clin Endocrinol Metab. 2022;107(7):E2882‐E2895. 35325149 10.1210/clinem/dgac178 PMC9202723

[11] Fleseriu M, Newell-Price J, Pivonello R, et al Long-term outcomes of osilodrostat in Cushing's disease: LINC 3 study extension. Eur J Endocrinol. 2022;187(4):531‐541. 35980235 10.1530/EJE-22-0317 PMC9513654

[12] Tabarin A, Haissaguerre M, Lassole H, et al Efficacy and tolerance of osilodrostat in patients with Cushing's syndrome due to adrenocortical carcinomas. Eur J Endocrinol. 2022:186:K1‐K4. 34905500 10.1530/EJE-21-1008

[13] Araujo-Castro M, García-Centeno R, González L, et al Real-world data on the efficacy and safety of osilodrostat in patients with Cushing's disease in Spain. J Clin Med. 2025;14(21):7575. 41226972 10.3390/jcm14217575 PMC12608593

[14] Laskowski G, Dzialach L, Maksymiuk-Kłos A, Witek P. Successful non-surgical treatment of bilateral macronodular adrenocortical disease with osilodrostat. Endokrynol Pol. 2025;76(6):674‐675. 41340349 10.5603/ep.106857

[15] Veloski C, Sturgeon A, Hallanger Johnson J. Prolonged adrenal insufficiency after failed cryoablation and osilodrostat for Cushing syndrome in nodular adrenal disease. JCEM Case Rep. 2025;3(6):luaf091. 40303510 10.1210/jcemcr/luaf091 PMC12037973

[16] Fleseriu M, Auchus RJ, Huang W, et al Osilodrostat treatment of Cushing syndrome in real-world clinical practice: findings from the ILLUSTRATE study. J Endocr Soc. 2025;9:bvaf046. 40226519 10.1210/jendso/bvaf046 PMC11986586

[17] Tabarin A, Bertherat J, Decoudier B, et al Real-world osilodrostat effectiveness and safety in nonpituitary Cushing syndrome. J Clin Endocrinol Metab. 2026;111 (5):1287‐1298. 41260612 10.1210/clinem/dgaf633 PMC13099221

[18] Motohashi K, Osawa N, Yamaji T, Tanioka T, Ibayashi H. Cushing syndrome. Nat Rev Dis Primers. 2025;11:3141‐3150. 5752303

[19] Fassnacht M, Johanssen S, Quinkler M, et al Limited prognostic value of the 2004 International Union Against Cancer staging classification for adrenocortical carcinoma: proposal for a Revised TNM Classification. Cancer. 2009;115(2):243‐250. 19025987 10.1002/cncr.24030

[20] Araujo-Castro M, Bancos I, Detomas M, et al Supplementary material for “International Real-world Study on Osilodrostat Efficacy and Safety in Adrenal Cushing Syndrome”. *Zenodo*. 2026. https://zenodo.org/records/18244071 10.1210/clinem/dgag115 PMC13368373 41824768

[21] Gadelha M, Snyder PJ, Witek P, et al Long-term efficacy and safety of osilodrostat in patients with Cushing's disease: results from the LINC 4 study extension. Front Endocrinol (Lausanne). 2023;14:1236465. 37680892 10.3389/fendo.2023.1236465 PMC10482037

[22] Castinetti F, Guignat L, Giraud P, et al Ketoconazole in Cushing's disease: is it worth a try. J Clin Endocrinol Metab. 2014;99(5):1623‐1630. 24471573 10.1210/jc.2013-3628

[23] Broersen LHA, Jha M, Biermasz NR, Pereira AM, Dekkers OM. Effectiveness of medical treatment for Cushing's syndrome: a systematic review and meta-analysis. Pituitary. 2018;21(6):631‐641. 29855779 10.1007/s11102-018-0897-z PMC6244780

[24] Dzialach L, Sobolewska J, Respondek W, Szamotulska K, Witek P. Cushing's disease: long-term effectiveness and safety of osilodrostat in a polish group of patients with persistent hypercortisolemia in the experience of a single center. Biomedicines. 2023;11(12):3227. 38137448 10.3390/biomedicines11123227 PMC10741245

[25] Araujo-Castro M, Garcia-Centeno R, González Fernández L, et al Efficacy and safety of osilodrostat in patients with ectopic Cushing’s syndrome. a real-world study in Spain. J Endocrinol Invest. 2025. Doi: 10.1007/s40618-025-02769-0 PMC13053458 41335191

[26] Ayala-Ramirez M, Jasim S, Feng L, et al Adrenocortical carcinoma: clinical outcomes and prognosis of 330 patients at a tertiary care center. Eur J Endocrinol. 2013;169(6):891‐899. 24086089 10.1530/EJE-13-0519 PMC4441210

[27] Fleseriu M, Pivonello R, Lacroix A, et al Osilodrostat dose impact on efficacy/safety in Cushing's disease: large, pooled analysis of LINC 2, 3, and 4. Eur J Endocrinol. 2025;193(5):506–5017. 10.1093/ejendo/lvaf207 41052284

[28] Fleseriu M, Pivonello R, Newell-Price J, et al Osilodrostat improves blood pressure and glycemic control in patients with Cushing's disease: a pooled analysis of LINC 3 and LINC 4 studies. Pituitary. 2025;28(1):22. 39863744 10.1007/s11102-024-01471-3 PMC11762609

[29] Nagendra L, Dutta D, Raizada N, Surana V, Selvan C, Bhattacharya S. Efficacy and safety of osilodrostat in managing Cushing's syndrome: a systematic review and meta-analysis. Indian J Endocrinol Metab. 2024;28(3):232‐238. 39086571 10.4103/ijem.ijem_260_23 PMC11288521

[30] Ferrari D, Bonaventura I, Simeoli C, et al Chronotherapy with once-daily osilodrostat improves cortisol rhythm, quality of life, and sleep in Cushing's syndrome. J Clin Endocrinol Metab. 2025;110(12):3525‐3537. 40172910 10.1210/clinem/dgaf206 PMC12623021

