# Correction to: “International real-world study on osilodrostat efficacy and safety in adrenal Cushing syndrome”

**DOI:** 10.1210/clinem/dgag216

**Published:** 2026-05-29

In the above-named article by Araujo-Castro M, Bancos I, Detomas M, Reincke M, Salehi M, Lu H, Altieri B, Kroiss M, Oettle M, Guerrero-Pérez F, Hanzu FA, García-Centeno R, Gónzalez-Fernandez L, Pasarón M, Gracia Gimeno P, Manzano Valero L, Castro Luna A, Iragaray Echarri A, Garcia-Argullo MDO, and Rivera Martínez WA (*J Clin Endocrinol Metab*. 2026; doi: 10.1210/clinem/dgag115) there was an error in the author list.

In the originally published article, in the author list, author Mahdi Salehi’s name was incorrectly published as “Salehi Mahdi.” In the corrected article, the authors’ name has been updated to “Mahdi Salehi.”

The article has been corrected online.

doi: 10.1210/clinem/dgag115

